# Patterned Well-Aligned ZnO Nanorods Assisted with Polystyrene Monolayer by Oxygen Plasma Treatment

**DOI:** 10.3390/ma9080656

**Published:** 2016-08-05

**Authors:** Hyun Ji Choi, Yong-Min Lee, Jung-Hoon Yu, Ki-Hwan Hwang, Jin-Hyo Boo

**Affiliations:** 1 Department of Chemistry, Sungkyunkwan University, Suwon 16419, Korea; hyunjee3807@hanmail.net (H.J.C.); lymin87@naver.com (Y.-M.L.); thank42@hanmail.net (J.-H.Y.); nercisus@naver.com (K.-H.H.); 2 Institute of Basic Science, Sungkyunkwan University, Suwon 16419, Korea

**Keywords:** ZnO nanorods, hydrothermal method, polystyrene monolayer, oxygen plasma treatment

## Abstract

Zinc oxide is known as a promising material for sensing devices due to its piezoelectric properties. In particular, the alignment of ZnO nanostructures into ordered nanoarrays is expected to improve the device sensitivity due to the large surface area which can be utilized to capture significant quantities of gas particles. However, ZnO nanorods are difficult to grow on the quartz substrate with well-ordered shape. So, we investigated nanostructures by adjusting the interval distance of the arranged ZnO nanorods using polystyrene (PS) spheres of various sizes (800 nm, 1300 nm and 1600 nm). In addition, oxygen plasma treatment was used to specify the nucleation site of round, patterned ZnO nanorod growth. Therefore, ZnO nanorods were grown on a quartz substrate with a patterned polystyrene monolayer by the hydrothermal method after oxygen plasma treatment. The obtained ZnO nanostructures were characterized by X-ray diffraction (XRD) and field-emission scanning electron microscope (FE-SEM).

## 1. Introduction

Zinc oxide is a semiconductor with a wide band gap of about 3.37 eV (at 300 K), a large free exciton-binding energy of 60 meV, high mechanical and thermal stabilities, and radiation hardness. To take advantage of these properties, nanostructures of ZnO have attracted both scientific and industrial interest owing to the possibility of being used in devices. Among the various nanostructures of ZnO, one-dimensional (1D) structures, such as nanowires, nanorods, nanotubes and nanoflowers, have attracted extensive attention over the past few years, due to their unique semiconducting and piezoelectric properties [1,2]. In particular, the alignment of ZnO nanorods into ordered nanoarrays has been extensively studied for use in various nanotechnology applications such as surface acoustic wave devices [3], gas sensors [4], UV lasers [5] and dye-sensitized solar cells [6].

Various methods have been developed and introduced to synthesize ZnO nanorods, including chemical vapor deposition (CVD), metal organic chemical vapor deposition (MOCVD) [7], vapor-liquid-solid (VLS) growth [8], sputtering [9] and pulsed laser deposition [10]. However, these growth methods require relatively high synthesis temperatures (>350 °C). In recent years, low-temperature approaches, such as the hydrothermal method, electro-deposition [11] and sol-gel processing [12], have become more important. Also, the hydrothermal method has the advantages of simple, large-scale product yield and sample uniformity at a relatively low temperature (60–100 °C) compared to the previously mentioned methods [13,14]. Therefore, the use of hydrothermal synthesis has allowed researchers to simply obtain the ZnO nanorods.

In order to apply ZnO nanorods to various sensing devices such as surface acoustic wave (SAW) sensors and quartz crystal microbalances (QCM), the nanorods have to be grown on a quartz substrate with piezoelectric properties. One of the problems associated with ZnO nanorods is the difficulty to achieve a well-ordered growth on the quartz substrate [15]. In order to overcome this problem, one approach for preparing well-ordered nanostructures with promising cost-effectiveness and high throughput is the colloidal crystal template approach using self-assembled mono-disperse polystyrene (PS) spheres as a template [16,17]. Also, ZnO nanorods have been fabricated on c-oriented ZnO seed layers by hydrothermal growth, particularly exploring the effects of annealing or plasma pre-treating the seed layer [18,19]. Such pre-treatments influence the density, size and surface defects of particles within the nucleating seed layer, which impact the subsequent nanorod growth via sequential reactions with OH^−^ and Zn^2+^ species. So, the oxygen plasma treatment prior to growing ZnO nanorods affects the nucleation. In this study, we investigate the patterned ZnO nanorods that were grown on a quartz substrate by partial oxygen plasma treatment using monolayers of PS spheres of various-diameters.

## 2. Results and Discussion

**Figure 1:**
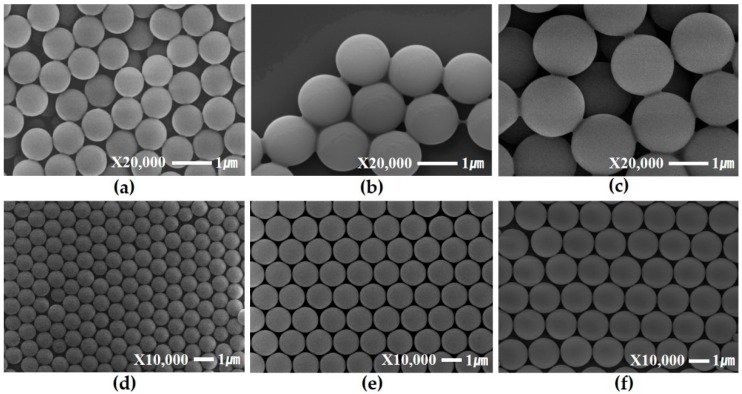
FE-SEM images of synthesized PS spheres with diameters of about (**a**) 800 nm; (**b**) 1300 nm; (**c**) 1600 nm and monolayers of (**d**) 800 nm; (**e**) 1300 nm and (**f**) 1600 nm.

Figure 1a–c shows the effect of the various reaction times on the polystyrene sphere size in the dispersion polymerization of styrene. Field-emission scanning electron microscopy (FE-SEM) images of synthesized PS spheres show that they have a highly uniform diameter. With increasing the reaction time, the size of the PS spheres increased proportionally (800 nm, 1300 nm and 1600 nm). Figure 1d–f shows the ordered PS sphere monolayer obtained through the conventional air-water interface-mediated method. Blowing air leads to the improvement of the crystal domain size by assisting recrystallization in the self-assembly process.

**Figure 2:**
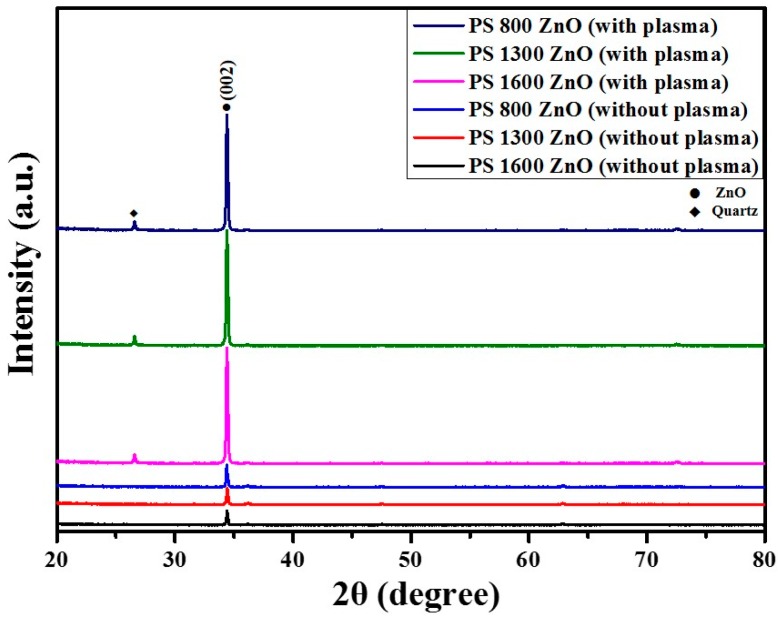
X-ray diffraction pattern of ZnO nanorod–assisted PS sphere monolayer arrays grown on a quartz substrate (with and without oxygen plasma treatment).

The crystal structure and the orientation of the as-grown nanorods array were revealed by X-ray diffraction (XRD) investigation. Figure 2 shows a typical XRD pattern of the vertically aligned ZnO nanorod–assisted PS sphere monolayer on the quartz substrate (with and without oxygen plasma treatment). The XRD pattern shows the hexagonal (wurtzite) structure of the ZnO nanorods (JCPDS No. 87-0713) and the quartz substrate (JCPDS No. 46-1045). The as-grown ZnO nanorod arrays show good crystallinity with a significantly intensified reflection of (002) at a 2θ value of 34.48°, indicating a preferential orientation of the nanorods with the c-axis perpendicular to the quartz substrate. In the case of ZnO nanorods without the oxygen plasma treatment, the intensity of the (002) peaks was very weak. On the other hand, the (002) reflection peaks of the ZnO nanorods with oxygen plasma treatment were higher intensity than without oxygen plasma treatment. The higher intensity of the (002) reflection peaks showed that the ZnO nanorods with the oxygen plasma treatment were in more vertical alignment than those without the oxygen plasma treatment.

**Figure 3:**
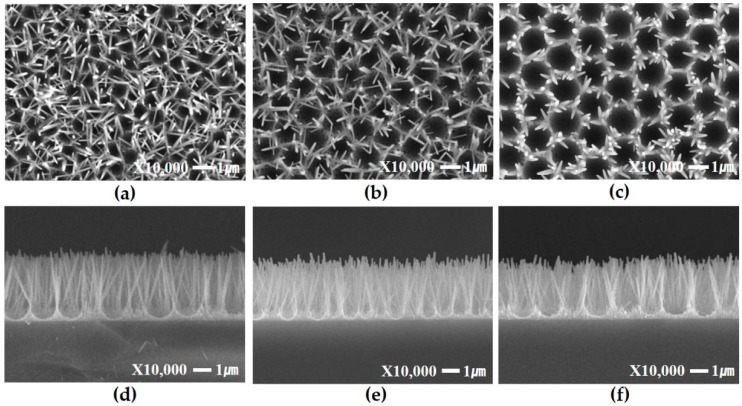
FE-SEM images of ZnO nanorod growth on quartz substrate using a PS monolayer template with oxygen plasma treatment. Diameters of polystyrene are (**a**) 800 nm; (**b**) 1300 nm; (**c**) 1600 nm and cross-section of (**d**) 800 nm; (**e**) 1300 nm and (**f**) 1600 nm, respectively.

Figure 3 shows a set of FE-SEM images corresponding to an array of round, patterned ZnO nanorods prepared by a hydrothermal method on a quartz substrate modified with an assembly of a polystyrene monolayer template with spheres that are 800 nm, 1300 nm and 1600 nm in diameter. This pattern is due to the elimination of the PS spheres after ZnO nanorod growth. The resulting length of the ZnO nanorods grown for 6 h at 90 °C in the same condition was approximately 2.6 μm. The oxygen plasma treatment is used to specify the nucleation site of the round, patterned ZnO nanorod growth.

**Figure 4:**
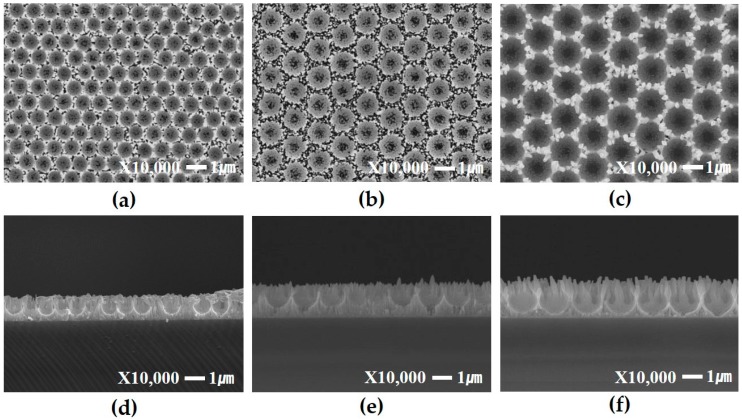
FE-SEM images of ZnO nanorod growth on quartz substrate by PS monolayer template without oxygen plasma treatment. Diameters of polystyrene are (**a**) 800 nm; (**b**) 1300 nm; (**c**) 1600 nm and cross-section of (**d**) 800 nm; (**e**) 1300 nm and (**f**) 1600 nm, resepectively.

As shown in Figure 4, the resulting length of the ZnO nanorods was approximately 740 nm. In normal cases (without oxygen plasma treatment), the seed layer/PS/solution interface has a higher surface energy than the unscreened surface (seed layer/solution interface). Therefore, there is greater possibility of forming the nucleation site at the seed layer/PS/solution interface than at the unscreened surface. Thus, ZnO nanorods did not completely accomplish a patterned structure. However, when the oxygen plasma treatment is used before growth, the unscreened areas are directly exposed to the plasma gas, causing an increase in the nucleation possibility and leading to preferential growth at the aperture site of the PS monolayer. In the (002) plane, the polar surface with –OH terminal groups contributes to vertically guiding these nanorods as a result of the strong bonding force, thus allowing a greater growth rate along this plane [20]. In other words, although the ZnO nanorods were grown for 6 h, the nanorods grew at a faster rate, compared to the case of those without the oxygen plasma treatment. Also, by using the PS sphere monolayer, oxygen plasma treatment not only was selectively carried out but also controlled the regular intervals of ZnO nanorods. Therefore, ZnO nanorods could be grown with selective and rapid growth rates.

## 3. Materials and Methods

### 3.1. Synthesis of Polystyrene

The polystyrene (PS) spheres with diameters of a few hundred nanometers were synthesized using dispersion polymerization. Briefly, the polymerization was carried out in an alcohol solvent system with 2,2-azobis(2-methylbutyronitrile) (AIBN, >98.0%, Tokyo Chemical Industry Co., Ltd., Tokyo, Japan) as an initiator. The flask was filled with 100 mL of ethyl alcohol (EtOH, Daejung Chemical Co., Ltd., Siheung, Korea), 0.1 g AIBN, 10 mL styrene (≥99.0%, Sigma-Aldrich, Munich, Germany) and polyvinyl pyrrolidone (PVP K-30, MW = 40,000 g/mol, Sigma-Aldrich) as stabilizer with magnetic stirring and nitrogen purging for 1 h. Then, the mixed solution was heated at 60 °C and sufficiently bubbled with nitrogen gas to eliminate the dissolved oxygen, which act as inhibitor in polymerization step. When the temperature of the reaction mixture remains steady at about 60 °C, the reaction proceeded. Detailed polymerization conditions are listed in Table 1.

### 3.2. Growth of Patterned ZnO Nanorods

Prior to the growth of the ZnO nanorods, Zinc acetate dehydrate (Zn(CH_3_COO)_2_·2H_2_O, Extra pure, Duksan, Ansan, Korea) was dissolved into 2-methoxyethanol (C_3_H_8_O_2_, >99.0%, Tokyo Chemical Industry Co., Ltd.) containing ethanolamine (C_2_H_7_NO, ≥99.0%, Sigma-Aldrich) and aged for two weeks with continuous stirring at room temperature. Before seed layer spin coating, the quartz substrate was cleaned with dilute hydrochloric acid, distilled water and ethanol for 10 min by sonication, respectively. After that, the quartz substrate was coated using the spin coating method (4000 rpm, 30 s) and annealed in a furnace at 500 °C for 30 min to secure the proper crystallinity for the growth of the nanorods.

Then the PS solution was slowly dropped on a water surface using a petri-dish which was tilted with respect to the water surface using micro-pipette. Then, SDS (Sodium dodecyl sulfate, ≥99.0%, Sigma-Aldrich) of surfactant was added on the water. On contact with the water, the PS spheres are spread and form a monolayer. Before being crystallized, air blowing was applied to the monolayer to induce the recrystallization of disordered PS spheres. Thus, the PS monolayer was transferred onto the seed coated substrate. PS layer coated substrate was dried under atmospheric condition. Then, the gap between ZnO nanorods was adjusted in three PS spheres sizes: 800 nm, 1300 nm and 1600 nm. Before ZnO nanorod growth, the oxygen plasma treatment (Covance-MPR, Femto Science Inc., Yongin, Korea) condition was 100 sccm, 100 W and 3 min for oxygen gas flow rate, plasma power, and time, respectively. For the growth of ZnO nanostructures on the patterned quartz substrate, zinc nitrate hexahydrate (Zn(NO_3_)_2_, 10 mM, >98.0%, Sigma-Aldrich) and hexamethlyene tetramine (HMTA, 10 mM, ≥99.0%, Sigma-Aldrich) were used as reacting agents in aqueous solution at room temperature for 2 h. Then ZnO nanorods were grown on the as-prepared patterned seed layer with the layer floating face-down in a Teflon bath. The reactions were carried out in an autoclave reactor (AID TR24, Aid Engineering, Hwaseong, Korea) at 90 °C for 6 h. After the reaction, the ZnO nanorod samples were calcined at 500 °C for 30 min to remove PS spheres. The crystal structure of the ZnO nanorods was characterized by X-ray diffraction (XRD, Bruker D8 Advance System, Dillerica, MA, USA) with filtered Cu (kα) radiation. The SEM images of the ZnO nanorods were measured by the field-emission scanning electron microscope (FE-SEM, JSM-7100F, JEOL, Tokyo, Japan).

## 4. Conclusions

In this work, the PS sphere monolayer–based patterning process was employed to control the shape, position, and orientation of the grown nanostructures through hydrothermal growth. Also, controlling the growth site structurally, the oxygen plasma treatment was confirmed to strongly affect the morphological structure of grown ZnO nanostructures. In addition, the comparative results of the ZnO nanorods with and without the oxygen plasma treatment were shown in this study; after the oxygen plasma treatment, the patterned ZnO nanorods became more highly ordered and grew more vertically.

Therefore, the alignment of ZnO nanostructures into ordered nanoarrays is expected to improve device sensitivity due to the increase in surface area to capture more gas particles. Moreover, this procedure could contribute to the development of optimized patterning of ZnO nanorod–based sensing devices, such as SAW sensors and QCM, and applied alignment of the sensitive structures can maximize the active areas of the sensors.

## Figures and Tables

**Table 1:** Polymerization conditions for the obtention of PS spheres.

Size (nm)	Solvent (mL)	Styrene (mL)	Initiator (g)	Stabilizer (g)	Reaction Time (h)	Reaction Temp. (°C)
PS 1600	EtOH 100	10	0.1	1.0	24	60
PS 1300	EtOH 100	10	0.1	1.0	16	60
PS 800	EtOH 100	10	0.1	1.0	8	60

## Abbreviations

The following abbreviations are used in this manuscript:

ZnO	Zinc oxide
PS	Polystyrene
FE-SEM	Field-emission scanning electron microscope
XRD	X-ray diffraction
rpm	Rounds per minute
sccm	Standard cubic centimeter per minute
